# Hippocampal Proteome of Rats Subjected to the Li-Pilocarpine Epilepsy Model and the Effect of Carisbamate Treatment

**DOI:** 10.3390/ph10030067

**Published:** 2017-07-30

**Authors:** José Eduardo Marques-Carneiro, Daniele Suzete Persike, Julia Julie Litzahn, Jean-Christophe Cassel, Astrid Nehlig, Maria José da Silva Fernandes

**Affiliations:** 1 Departamento de Neurologia e Neurocirurgia, Disciplina Neurociência, Escola Paulista de Medicina - Universidade Federal de São Paulo, São Paulo-SP 04039-032, Brazil; edumarques83@gmail.com (J.E.M.-C.); daniele_persike@protonmail.com (D.S.P.); julie.litzahn@gmail.com (J.J.L.); 2 Unistra, Laboratoire de Neurosciences Cognitives et Adaptatives—Université de Strasbourg, Faculté de Psychologie, 67000 Strasbourg, France; jcassel@unistra.fr; 3 CNRS, UMR 7364, LNCA, 12 rue Goethe, 67000 Strasbourg, France; 4 INSERM U 1129 “Infantile Epilepsies and Brain Plasticity”, 75015 Paris, France; nehliga@unistra.fr; 5 Université Paris Descartes, Sorbonne Paris Cité; CEA, 91990 Gif sur Yvette, France

**Keywords:** carisbamate, temporal-lobe epilepsy, hippocampus, proteomics, brain activity

## Abstract

In adult rats, the administration of lithium–pilocarpine (LiPilo) reproduces most clinical and neuropathological features of human temporal lobe epilepsy (TLE). Carisbamate (CRS) possesses the property of modifying epileptogenesis in this model. Indeed, about 50% of rats subjected to LiPilo status epilepticus (SE) develop non-convulsive seizures (NCS) instead of motor seizures when treated with CRS. However, the mechanisms underlying these effects remain unknown. The aim of this study was to perform a proteomic analysis in the hippocampus of rats receiving LiPilo and developing motor seizures or NCS following CRS treatment. Fifteen adult male Sprague–Dawley rats were used. SE was induced by LiPilo injection. CRS treatment was initiated at 1 h and 9 h after SE onset and maintained for 7 days, twice daily. Four groups were studied after video-EEG control of the occurrence of motor seizures: a control group receiving saline (CT *n* = 3) and three groups that underwent SE: rats treated with diazepam (DZP *n* = 4), rats treated with CRS displaying NCS (CRS-NCS *n* = 4) or motor seizures (CRS-TLE *n* = 4). Proteomic analysis was conducted by 2D-SDS-PAGE. Twenty-four proteins were found altered. In the CRS-NCS group, proteins related to glycolysis and ATP synthesis were down-regulated while proteins associated with pyruvate catabolism were up-regulated. Moreover, among the other proteins differentially expressed, we found proteins related to inflammatory processes, protein folding, tissue regeneration, response to oxidative stress, gene expression, biogenesis of synaptic vesicles, signal transduction, axonal transport, microtubule formation, cell survival, and neuronal plasticity. Our results suggest a global reduction of glycolysis and cellular energy production that might affect brain excitability. In addition, CRS seems to modulate proteins related to many other pathways that could significantly participate in the epileptogenesis-modifying effect observed.

## 1. Introduction

Recurrent spontaneous motor seizures are characteristic of temporal lobe epilepsy (TLE) [1]. They are associated with significant neuronal loss and morphological alterations which affect mainly mesial temporal structures, such as hippocampal formation and amygdala [1,2,3]. The animal model of TLE induced by pilocarpine (Pilo) reproduces the main clinical and pathophysiological features of human TLE, including mossy fiber sprouting, hippocampal sclerosis and gliosis [4,5]. In this model, SE is followed by a latent period of 7–44 days until the appearance of spontaneous recurrent motor seizures [4].

Approximately 30% of patients with MTLE present refractory seizures, despite numerous antiepileptic drugs (AEDs) commercially available. Carisbamate (CRS, RWJ-333369; S-2-*O*-carbamoyl-1-o-chlorophenyl-ethanol) is a new drug that possesses the property to dramatically modify epileptogenesis in an experimental model of TLE [6,7,8]. These findings were observed when CRS was applied starting 1 h after SE induced by lithium–pilocarpine (LiPilo). In brief, CRS induces widespread neuroprotection and, most importantly, a disease- and insult-modifying effect in a fraction of treated animals [8]. Some animals that underwent LiPilo SE developed non-convulsive seizures (NCS) instead of the convulsive seizures commonly observed in this model. These NCS recall absence seizures characterized by behavioral arrest accompanied by bilateral synchronous spike-and-wave-discharges [9,10]. In addition, this sub-population of rats presents a cognitive profile similar to that of control rats while rats with motor seizures are dramatically impaired [11,12]. However, the mechanisms underlying these CRS-induced changes in the nature of LiPilo-induced epilepsy are still unknown.

Previous studies looking at direct changes induced by this drug have shown that CRS may inhibit voltage-gated sodium channels, thereby reducing action potential discharges and acting on neuronal excitability [13]. It was also shown that CRS inhibits alpha-amino-3-hydroxy-5-methyl-4-isoxazolepropionic acid (AMPA) and N-Methyl-*D*-aspartic acid (NMDA) excitatory post-synaptic potentials, reducing glutamatergic transmission [14]. Finally, CRS affects also the serotonergic system by increasing the tonic activation of somatodendritic 5-HT_1A_ receptors, which leads to an inhibitory action over hippocampal pyramidal cells [15]. In the same work, it was shown that CRS also acts on noradrenergic and dopaminergic systems [15]. 

These mechanisms suggest that CRS can reduce neuronal excitability by affecting various neurotransmitter systems and pathways synergistically. However, even if these results reveal potential mechanisms of action of CRS, the effect of CRS over voltage-gated sodium channels, AMPA and NMDA excitatory post-synaptic potentials as well as on serotonergic, noradrenergic and dopaminergic systems do not explain the fact that in the chronic period of the LiPilo model about half the rats treated with CRS do display NCS, with characteristics similar to absence seizures, instead of convulsive seizures [8].

Herein, we explored changes occurring at two months after SE in the LiPilo model, by comparing rats displaying convulsive seizures (treated or not with CRS), rats displaying NCS, and control rats. We performed a proteomic study on the hippocampus of those rats to identify protein changes that could help understanding why CRS is able to modify the expression of the disease. Our goal was to perform a hippocampal proteome highlighting changes that might be the cause or consequence of this disease-modifying feature. We hope that our study will help to identify new avenues of research for future studies. 

## 2. Results

**Figure 1:**
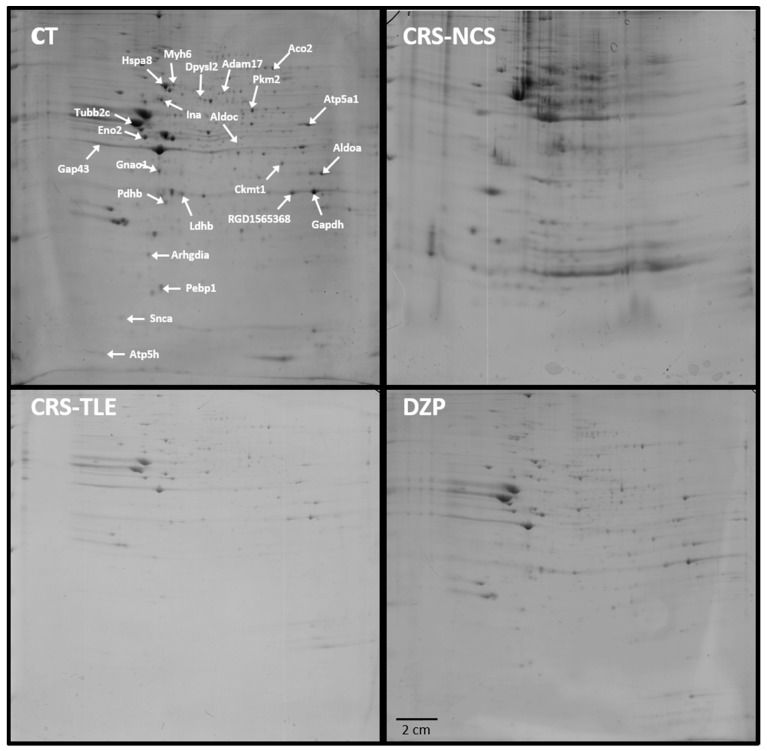
SDS-PAGE gel taken from a control (CT) rat showing the localization of all 24 spots of interest in a gel of the CT group (left upper quadrant). The other experimental groups studied were lithium–pilocarpine (LiPilo) rats treated with carisbamate (CRS) that subsequently displayed limbic seizures (CRS–TLE, left lower quadrant), LiPilo rats treated with CRS that subsequently displayed non-convulsive seizures (CRS-NCS, right upper quadrant), and LiPilo rats treated with diazepam (DZP, right lower quadrant).

Using two-dimensional separations of hippocampal homogenates, we could identify 24 proteins differentially expressed among the four experimental groups (Figure 1 and Table 1). The identification data was submitted to the PeptideAtlas (Id: PASS00899), a database that is part of the ProteomeXchange Consortium.

### 2.1. Proteomics

The results revealed an increase of Dypsl2, Hspa8 and Gap43, a reduction of Gnai1, Pebp1, and Pdhb, in the DZP as compared to the CT group. There was also an up-regulation of Atp5d, Pdhb and Ldhb, and a down-regulation of Aco2, Adam17, Aldoa, Atp5a1, Ckmt1, Gapdh, Gnai1, Myh6, Pkm, Arhgdia, and Tubb2c in CRS-NCS as compared to CT rats. Furthermore, an increased level of Eno2 and Gap43, and decreased level of Ina, Snca, Gnai1, Ldhb, and Pebp1 were observed in CRS–TLE as compared to CT animals. The comparison between the CRS–NCS and DZP group showed up-regulation of Snca, Atp5d, Eno4, Ldhb, Pebp1, and Pdhb, and down-regulation of Aco2, Adam17, Aldoa, Aldoc, Atp5a1, Ckmt1, Dpysl2, Gapdh, Hspa8, Myh6, Gap43, Pkm2, Arhgdia, and Tubb2c in CRS–EA rats. The CRS–TLE group showed up-regulation of Eno2, and Gap43, and down-regulation of Aldoc, Dpysl2, Hspa8, and Ldhb as compared to DZP. Up-regulation of Snca, Atp5d, Eno4, Ldhb, Pebp1, and Pdhb, and down-regulation of Aco2, Adam17, Eno2, Gapdh, Myh6, Gap43 Pkm2, and Arhgdia were recorded in CRS–NCS as compared to CRS–TLE rats. The identified spots are shown in Figure 1 representing a gel from a CT rat.

### 2.2. Interactome

**Figure 2:**
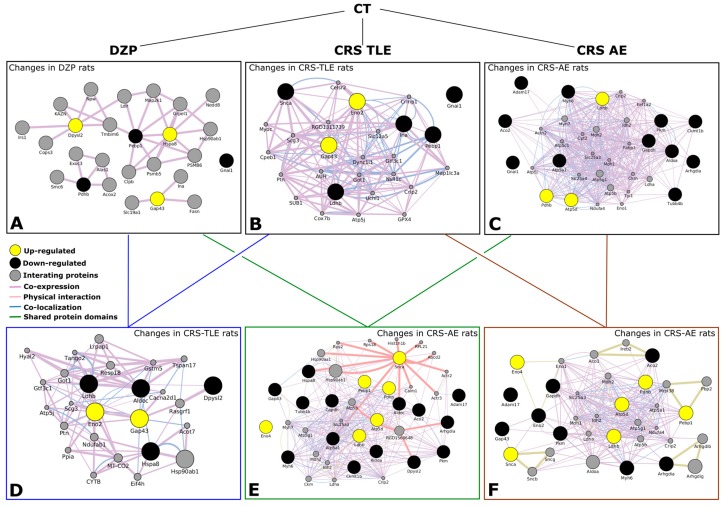
Schematic representation of the interactome obtained from relevant comparisons between groups. (**A**) Changes in DZP compared to CT rats; (**B**) Changes in CRS–TLE compared to CT rats; (**C**) Changes in CRS–NCS compared to CT rats; (**D**) Changes in CRS–TLE compared to DZP rats; (**E**) Changes in CRS–NCS compared to DZP rats; (**F**) Changes in CRS–NCS compared to CRS–TLE rats. Proteins up-regulated are indicated in yellow; proteins down-regulated are indicated in black; interacting proteins are indicated in gray. The size of the gray spots indicates the weight of the interaction. The lines indicate the type of interaction according to the color code indicated in the Figure.

Using the GENEmania system we were able to identify 74 biological functions for our proteins of interest, and 20 other proteins that have some kind of interaction with these proteins. Using the GO ID’s generated by the GENEmania system and Cytoscape software; we could put all this information together, which facilitated data analysis. Figure 2 shows an interaction map for all relevant comparisons. In each case, 20 proteins with the higher interaction level were included. The comparison between DZP vs. CT rats (Figure 2A) shows three up-regulated proteins and three down-regulated proteins in DZP rats. Two up-regulated and 5 down-regulated proteins were included in Figure 2B for the interaction map of the comparisons between CRS–TLE and CT group. Figure 2C shows the interaction map including the three up-regulated and the 11 down-regulated proteins when comparing CRS–NCS to CT rats. The comparison between CRS–TLE and DZP rats is shown in Figure 2D, which includes two up-regulated and four down-regulated proteins. The interaction map for the six up-regulated and fourteen down-regulated proteins found when comparing CRS-NCS to DZP rats is represented in Figure 2E. Finally, the comparison between CRS-NCS and CRS-TLE rats is represented in the Figure 2F and includes six up-regulated and eight down-regulated proteins. 

**Figure 3:**
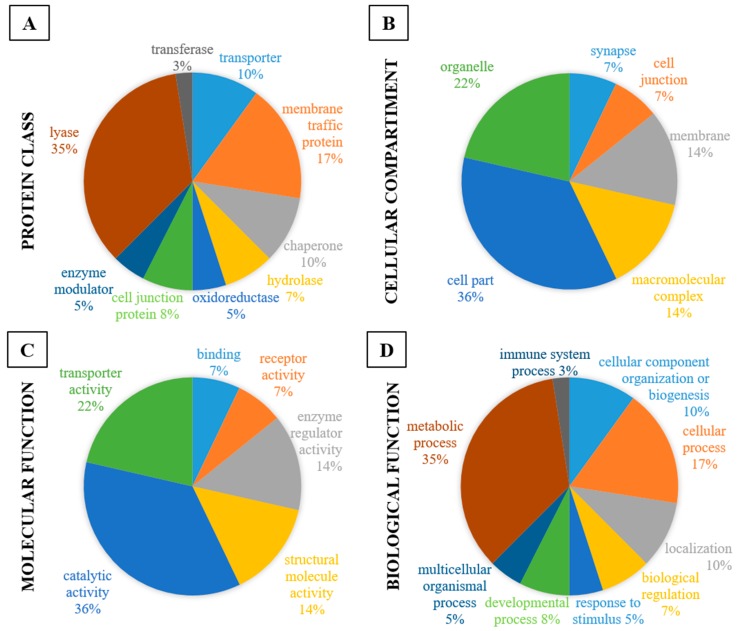
Schematic profile indicating the proportion (%) of the proteins of interest in each category: Protein class (**A**); cellular compartment (**B**); molecular function (**C**) and biological function (**D**).

The proteins of interest revealed by the proteomic study include proteins of nine different protein classes (Figure 3A), six different cellular compartments (Figure 3B), six different molecular functions are related to our proteins of interest (Figure 3C) and nine biological functions as shown in Figure 3D. 

As can be observed in Figure 3A, the majority of identified proteins are lyases (35%) or membrane traffic proteins (17%) but there are also chaperones (10%), transporters (10%), cell junction proteins (8%), hydrolases (7%), enzyme modulators (5%), oxidoreductases (5%) and transferases (3%). Regarding the cellular compartment of identified proteins, 36% are located in the cell partition, 22% in organelles, 14% are macromolecular complexes, 14% are membrane proteins, 7% are proteins located in synapses and 7% are cell junction proteins (Figure 3B). The interactome also revealed that identified proteins are mainly related to catalytic activity (36%) and transporter activity (22%), but we also found structural molecules (14%), regulator enzymes (14%), receptors (14%) and binding proteins (7%) (Figure 3C). Finally, in the list of biological functions related to the proteins of interest, 35% are related to metabolic functions, 17% to cellular processes. About also 10% of proteins are classified according to localization, 10% are linked to biogenesis or component organization, 8% are developmental proteins, 7% are related to biological regulations, 5% are linked to multicellular organismal processes, 5% to response to stimulus and 3% are related to the immune system (Figure 3D). 

**Figure 4:**
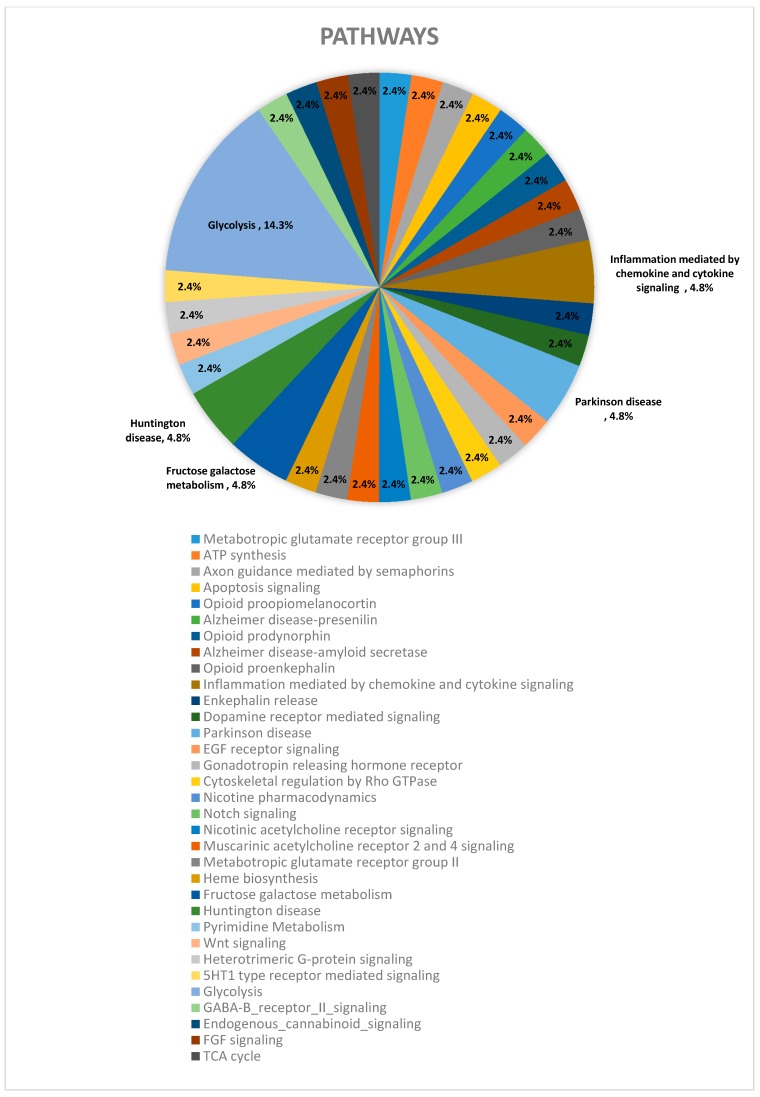
Schematic representation of the pathways identified in the interactome, indicating the proportion (%) of proteins of interest related to each pathway.

In addition to these global findings, we generated interactome crossing the groups, to know more details about protein classes, cell compartment, molecular and biological functions which play the proteins of interest (Table 2). The same strategy was used to know about the pathways in which the proteins of interest are involved in each group (Figure 4) and crossing the groups (Table 3).

The interactome analysis also revealed that the proteins identified in the study, are mainly related to glycolysis (14%) and to inflammation processes mediated by chemokine and cytokine signaling (5%). We also found proteins associated to Huntington’s (5%) and Parkinson’s disease (5%) and also associated to fructose and galactose metabolism (4.80%). In addition, minor changes (2%) were also observed in several other pathways.

### 2.3. Western Blot Confirmation

**Figure 5:**
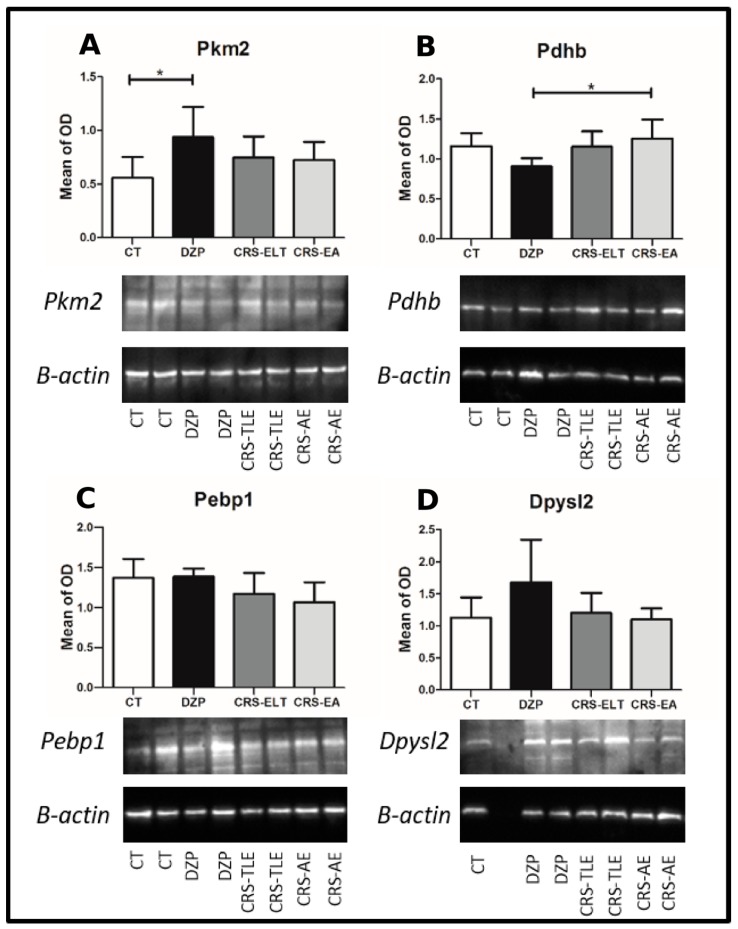
Expression (mean optical density (OD) normalized with β-actin) of four proteins of interest quantified by Western blotting. (**A**) Pyruvate kinase isoform (M2–Pkm2), (**B**) Pyruvate dehydrogenase subunit E1 (Pdhb), (**C**) Phosphatidylethanolamine binding protein *1* (Pebp1) and (**D**) Dihydropyrimidinase like protein *2* (Dpysl2). An antibody raised against β-actin was used as loading control. Samples derive from the same experiment and gel images shown were not cropped. * *p* < 0.05.

The Western blotting data pointed to an up-regulation of Pkm2 in the DZP vs. CT group (F(3, 20) = 3.46; *p* = 0.035; BS *p* = 0.049—Figure 5A), and a higher level of Pdhb in CRS-NCS vs. DZP group (F(3, 20) = 4.57; *p* = 0.013; BS *p* = 0.018—Figure 5B). However, no statistical intergroup difference was observed in the level of Pebp1 (F(3, 20) = 3.28; *p* = 0.042; BS *p* = 0.051) and Dypsl2 (F(3, 20) = 2.94; *p* = 0.057; BS *p* = 0.124) (Figure 5C,D).

## 3. Discussion

CRS is an anticonvulsant drug that produces broad neuroprotection and possesses an interesting disease-modifying effect in an animal model of TLE. Regarding the disease-modifying effect, a subpopulation of rats treated with CRS, developed NCS instead of the convulsive seizures commonly observed in this model [8]. These NCS are very similar to the spikes-and-wave discharges observed in animal models for absence epilepsy, and which are the main feature of absence epilepsy, accompanied by behavioral arrest [10,16]. However, one needs to keep in mind that given that CRS treatment started at 1 h after SE onset, both an insult- and disease-modifying effect might underlie the change in the nature of the disease due to CRS treatment.

To highlight changes related to these insult- and disease-modifying effects, in the present study, we analyzed the hippocampal proteome of all groups that underwent LiPilo SE compared to saline-exposed animals. There is a growing number of studies using proteomic techniques to identify protein changes and biomarkers for epilepsy [17,18,19,20]. Some of these studies have used hippocampal tissue surgically removed from patients with refractory epileptic seizures [21,22]; others have used tissue from animal models of TLE [23,24]. The proteomics based on two-dimensional electrophoresis allowed us to get a visual map of the hippocampal proteome. It offers advantages over methods of separation in which protein digestion is requested before separation [17].

In the present study, we identified the differential expression of proteins related to different biological/molecular functions and pathways (see Figure 3 and Figure 4). Briefly, these proteins are mainly related to neuronal development and plasticity, glycolysis and energy production, and neuronal excitability.

### 3.1. Neuronal Development and Plasticity

We observed alterations in proteins that are related to neuronal development and plasticity. This was the case of Phosphatidylethanolamine-binding protein *1* (Pebp1), which was down-regulated in both groups displaying motor seizures (DZP and CRS–TLE) when compared to CT and CRS–NCS groups. Pebp1, an endogenous serine protease inhibitor, exerts inhibitory activity against several serine proteases including thrombin, neuropsin, and chymotrypsin (not trypsin), tissue-type plasminogen activator, and elastase [25]. Many processes are regulated by serine proteases, including tissue homeostasis, neuronal growing [26], cell migration [27], cell death [28], and cell survival [29]. Therefore, the “normalization” of Pebp1 protein observed in the CRS–NCS group could reflect improved tissue homeostasis in these rats, while in epileptic rats its down-regulation could explain several deregulations and dysfunctions. 

The Dihydropyrimidinase-related protein *2* (Dpysl2) was up-regulated in DZP rats, while both groups treated with CRS had a level similar to that of the CT group. Interestingly, this protein was also found up-regulated in human hippocampal tissue from patients with TLE when compared to control subjects [30]. Dpysl2 has an essential role in development and neuronal polarity, axon growth and guidance, and in neuronal migration [31]. Thus, the increase in expression of this protein in the hippocampus of DZP rats might be related to neurogenesis and synaptic reorganization that is classically observed in TLE. On the other hand, its “normalization” in CRS-treated rats (both CRS–TLE and CRS–NCS) suggests a reduction of tissue reorganization that can go as far as the prevention of convulsive seizures in a sub-population of rats.

The protein Neuromodulin (Gap43) was found up-regulated in rats with convulsive seizures (DZP and CRS–TLE) compared to CT and CRS–NCS. Gap43 has been associated to structural plasticity, synaptic reorganization as well as neural development [32]. In addition, Gap43 seems to be linked with mossy fiber sprouting in experimental models of epilepsy [33]. This protein is related to memory and learning [34,35,36]. However, while moderate overexpression of Gap43 can enhance memory, excessive overexpression may lead to memory dysfunction [34]. Our results show a similar level of Gap43 in CRS–NCS compared to CT rats while it was increased in the DZP and CRS–TLE groups. Given the properties of GAP43 in learning abilities, these data are in agreement with strongly degraded cognitive abilities in DZP and CRS–TLE rats while cognitive performance of CRS–NCS remains remarkably close to that of CT rats [11,12].

Finally, the Rho GDP-dissociation inhibitor *1* (Arhgdia or Rhogdi1) was reduced in CRS–NCS compared to the three other groups. This protein is associated with neural development, axonal and dendritic growth, anti-apoptosis effects, cell movement and negative regulation of cell adhesion [37,38]. Moreover, Arhgdia has also been attributed a chaperone function, protecting multiple Rho proteins from proteolytic degradation [37,39]. 

Altogether, these results suggests that CRS treatment exerts a modulatory role in Pebp1, Dpysl2, Gap43 and Arhgdia, which could act on/reflect plasticity-related events commonly observed in animals presenting convulsive seizures but not in animals displaying NCS.

### 3.2. Glycolysis and Energy Production 

**Figure 6:**
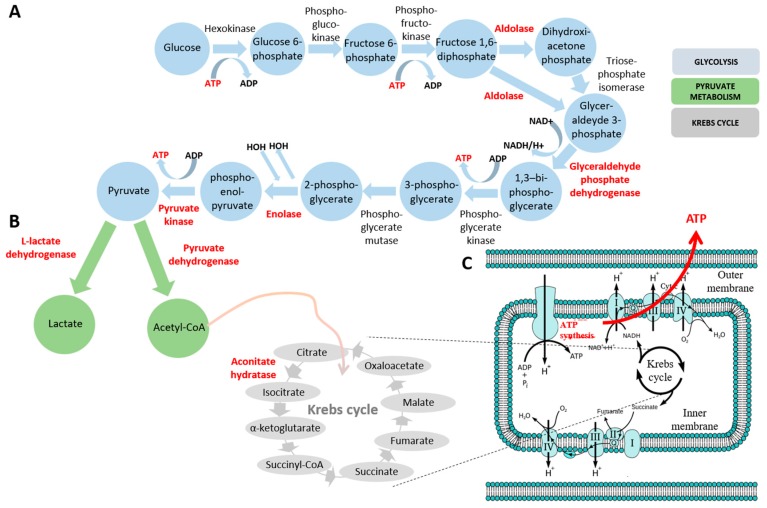
Schematic representation of the main alterations in metabolic pathways revealed by proteomic analysis. The color code of the different pathways is the following: glycolysis (**A**), pyruvate metabolism (**B**), and Krebs cycle and oxidative phosphorylation (**C**). Affected proteins are indicated in red and bold.

Interestingly, the present study revealed that almost one-third of altered proteins are related to glycolysis and energy supply (Figure 6). The whole glycolytic process comprises 10 reactions catalyzed by cytosolic enzymes during which a glucose molecule is oxidized to produce two molecules of pyruvate (Figure 6). Therefore, the reduction of Aldoa, Gapdh, and Pkm2 suggests a global reduction of the glycolytic process in rats treated with CRS and displaying NCS as compared to rats with convulsive seizures. Carbohydrates and lipids are the main classes of molecules involved in oxidative metabolism [40] and glycolysis is the main source of cellular energy, being essential for brain and red blood cells [40]. In turn, pyruvate, the final product of glycolysis, can be metabolized into acetyl-CoA by pyruvate dehydrogenase (PDH), in particular the subunit alpha E1 of pyruvate dehydrogenase (Pdhb). This subunit is responsible for the activation/deactivation of the PDH complex [41] and is the first link between glycolysis and the tricarboxylic acid cycle (TCA). On the other hand, pyruvate can be converted into lactate by the action of L-lactate dehydrogenase (LDH). These enzymes were up-regulated in the CRS–NCS group. However, given the global reduction of expression of glycolysis-related proteins, the up-regulation of both enzymes, Pdhb and LDH observed in the CRS–NCS group may be a compensatory mechanism allowing a sufficient flow of pyruvate into the TCA. 

The acetyl-CoA, after entering the TCA, is converted into citrate by the action of the enzyme Aco2. The reduction of Aco2 in CRS–NCS rats suggests a possible reduction of NADH^+^, H^+^ and GTP levels. NADH^+^ is generated by glycolysis and TCA transfers of electrons to the cytochromes [42]. This NADH^+^ is used in the synthesis of ATP from ADP + Pi, in a proportion of 3 ATP molecules for each NADH^+^. We also found in the CRS–NCS group a reduction of Atp5a1, a subunit of mitochondrial complex V at which level the synthesis of ATP from ADP takes place in the presence of a membrane proton gradient [42].

Neuronal excitability is closely related to energy metabolism. First, the maintenance of neuronal activity incurs a significant energy demand, which requires very active cellular metabolism [43]. In addition, neurons in which the primary function is not primarily related to metabolism can also alter their level of excitability in response to metabolic changes [43]. There is also some molecular link between metabolism and neuronal excitability such as the nearby ATP-sensitive potassium (K_ATP_) channels that are activated by sub-membrane ATP consumption by ionic pumps [44,45]. A reduction of the glycolytic pathway is also observed in the ketogenic diet, a treatment used to control drug-resistant seizures [43].

### 3.3. Neuronal Excitability

In addition to these proteins related to glycolysis and cellular respiration, other proteins might also have an impact on neuronal excitability. It is the case of the Heat shock cognate 71 kDa protein (Hspa8), Alpha-synuclein (Snca) and Tubulin beta-2C chain (Tubb2c). The Hspa8, which we found up-regulated in DZP rats but not in both groups treated with CRS (CRS–TLE and CRS–NCS) is a chaperone protein that has an important role in heat-induced stress. This protein also interacts with the intracellular CHL1 domain (a cell adhesion protein), being internalized and directed to synaptic vesicles [46]. In fact, Hspa8 has a role in synaptic vesicle biogenesis and is responsible, together with the auxilin protein, of the disassembly of the clathrin protein through an ATP-dependent reaction, therefore controlling the release of vesicles [47]. The fact that Hspa8 is increased only in the DZP group suggests an altered release of neurotransmitters such as GABA and glutamate, but also monoamines, affecting the excitatory imbalance characteristic of epilepsy. In rats treated with CRS, whatever the epileptic fate, TLE or NCS, the level of this protein was not affected. 

The Snca, which was down-regulated in both groups displaying convulsive seizures (DZP and CRS–TLE), but not in CRS–NCS rats, is an abundant protein in the brain, found mainly in presynaptic terminals. Different neurodegenerative diseases show alterations in this protein [48,49]. This protein is able to form pores on ion channels in the membrane with direct consequence on conductivity [50]. An important role for this protein has been suggested in maintenance of the supply of synaptic vesicles in presynaptic terminals [51]. Snca plays also a role in the regulation of dopamine transporters [52], and dopamine biosynthesis by reducing the activity of tyrosine hydroxylase, a rate-limiting dopaminergic enzyme [53]. Hence, alterations of Snca observed in rats displaying TLE could potentially affect the dopaminergic system.

Finally, we found also an increased level of Tubulin beta-2C chain (Tubb2c) in the DZP group, but levels were reduced in the CRS-TLE and CRS-NCS groups. The Tubb2c has an important role in protein folding, a process in which a protein reaches its functional conformation. In addition, this protein also acts in the axonal vesicle transport and Glut4 [54]. 

### 3.4. Pathways Observed in the CRS-NCS Group

Our interactome analysis revealed that the activation of pathways such pyruvate metabolism, TCA cycle were observed only in comparisons made against the group CRS–NCS supporting the role of proteins related to energy production and the outcome observed in this group. In addition, all comparisons made against CRS–NCS also revealed the involvement with pathways such as cytoskeletal regulation by Rho GTPase, Notch signaling, nicotinic acetylcholine receptor signaling.

In the Panther database, the cytoskeletal regulation by the Rho GTPase pathway implies that cell moves and shape changes need rearrangements at the level of filaments that are regulated by Rho GTPase (P00016—www.pantherdb.org). Interestingly, it was observed that rhoA knockout animals develop epileptic seizures during the first postnatal month [55,56], supporting the role of this protein in convulsive seizures.

Notch is a trans-membrane receptor that mediates local cell–cell communication as well as in the coordination of the signaling cascade (P00045—www.pantherdb.org). Traditionally, the Notch pathway is associated to the regulation of neural stem cells; however, recent evidence suggests different roles for Notch signaling in mature brain [57], and mainly an association with neuronal discharges. Noteworthy, Notch signaling was found activated in a model of TLE and in human epileptogenic tissues [58].

The nicotinic acetylcholine receptor signaling also has a role in epilepsy. Indeed, it was identified in a genetically transmissible form of epilepsy (frontal-lobe epilepsy) that is associated with a mutation in the gene CHRNA4. This gene encodes a subunit of the nicotinic acetylcholine receptor. Interestingly, polymorphisms at the level of this gene can disrupt the normal functioning of these receptors and may provoke the imbalance between excitation and inhibition and cause seizures [59,60].

Thus, it appears that the hippocampus of CRS–NCS rats has undergone a wide array of molecular changes compared to the DZP and CRS–TLE rats which might partly underlie the change in their epileptic fate. 

## 4. Materials and Methods

**Animals**: Thirty-nine adult male Sprague–Dawley rats (355 ± 31 g) provided by the University of São Paulo were housed under controlled conditions (22 ± 1 °C, 12/12 h light/dark cycle, lights on at 7:00 a.m.) with water and food *ad libitum*. The ethics research committee of the Federal University of São Paulo (CEP N° 2072/11) approved all experiments. Efforts were made to minimize pain or discomfort of animals. The experiments were performed following the principles outlined in the ARRIVE (Animal Research: Reporting of In Vivo Experiments) guidelines and the Basel declaration (http://www.basel-declaration.org). The 3R concept (Replacement, Refinement and Reduction of Animals in research) has been considered when planning the experiments. 

**SE Induction and CRS treatment**: The protocol used were identical to our previous studies [8,11,12]. In brief, rats were injected i.p. with 127 mg/kg lithium chloride. About 18 h later they received methylscopolamine (1 mg/kg s.c., Sigma-Aldrich, St. Louis, MO, USA) in order to limit the undesirable peripheral effects of pilocarpine. Thirty min later we induced the SE by a administration of pilocarpine (25 mg/kg s.c., Sigma-Aldrich). The control group also received lithium chloride but saline instead of pilocarpine. Then, 1 and 9 h after SE onset, rats were randomly administered with diazepam (DZP, 2.5 and 1.25 mg/kg i.m., respectively, Roche, Meylan, France) or CRS (90 mg/kg i.p., Johnson & Johnson Research & Development, L.L.C., Raritan, NJ, USA) dissolved in 45% hydroxypropyl-β-cyclodextrin (Acros Organics, Geel, Belgium). DZP administered at low doses enhanced survival of rats without modifying SE characteristics [61]. Over the following six days, the CRS-treated groups received CRS injections (90 mg/kg, s.c.) twice daily. Untreated group received saline instead of CRS. Rats were euthanized 2 months after SE induction. 

**Video monitoring**: To avoid molecular alterations (such inflammation) due to electrode implantation, we performed the monitoring for seizure identification only by using a video-recording system. Starting one week after SE, video monitoring (24 h/7 days) was performed over a 2-month period in order to assess seizure onset. Two groups of rats were obtained after CRS treatment, one displaying convulsive seizures and the other one displaying non-convulsive seizures. We used the Racine scale to classify convulsive seizures and rats presenting at least one stage III motor forelimb clonus were used in the study [62]. As we showed previously, CRS-treated rats without convulsive seizures and overt behavioral changes display absence-like spike-and-wave discharges (NCS) [8].

**Experimental groups**: Four experimental groups were used: (1) CT—a control group receiving lithium chloride and saline; (2) DZP—rats that underwent SE and were treated with DZP 1 h after SE onset; (3) CRS–TLE—rats that underwent SE, were treated with CRS and developed limbic seizures; and (4) CRS–NCS—rats that underwent SE, were treated with CRS and developed non-convulsive seizures. 

**Sample preparation**: Two-dimensional SDS-PAGE was applied. Fifteen animals were used in this experiment (3 CT; 4 CRS–NCS; 4 CRS–TLE and 4 DZP). Two months after SE, rats were euthanized by decapitation and right and left hippocampi were dissected out, weighed and stored at −80 °C. Briefly, hippocampal samples were homogenized in lysis buffer and the protein concentration was calculated using the Bradford method [63]. After protein precipitation, an IPG strip with linear pH (3–10) was used for the first dimension separation by isoelectric-focalization (IEF). After IEF, a polyacrylamide gel (SDS-PAGE) was used for the second dimension separation. At the end of the run, gels were stained with Comassie blue and then scanned by a densitometer, normalized to the background and analyzed in the PDQuest 2D-gel software for quantification. Spots of interest were cut from the gel to proceed to mass spectrometry identification. For more liability, we proceeded to the identification in duplicates (each spot of interest was cut from two different experimental groups). After being cut, spots were washed three times in a 100 mM solution containing 50% of acetonitrile and 50 mM of (NH_4_)_2_CO_3_. After removing the supernatant, each spot was incubated with trypsin (200 ng of trypsin diluted in 50 mM (NH_4_)_2_CO_3_, 10% acetonitrile in H_2_O) overnight at 37 °C, for protein digestion. The addition of formic acid 2% during 1 h was used to stop the digestion. Then, the supernatant was transferred to a new vial that was used for identification in the mass spectrometer.

**Mass spectrometry analyses**: Peptides mixtures were separated by C18 (100 µm × 100 mm) RP-nanoUPLC (nanoAcquity, Waters, Milford, MA, USA) coupled with a Q-Tof Premier mass spectrometer (Waters) with nanoelectrospray source at a flow rate of 0.6 µL/min. The gradient was 2–30% acetonitrile in 0.1% formic acid over 5 min for the digested proteins. The nanoelectrospray voltage was set to 3.5 kV, a cone voltage of 30 V and the source temperature was 80 °C. The instrument was operated in the ‘top three’ mode, in which one MS spectrum is acquired followed by MS/MS of the top three most-intense peaks detected. After MS/MS fragmentation, the ion was placed on exclusion list for 20 s and for the analysis of endogenous cleavage peptides, a real time exclusion was used. The spectra were acquired using software MassLynx v.4.1 (Milford, MA, USA) and the raw data files were converted to a peak list format (mgf) without summing the scans by the software Mascot Distiller v.2.3.2.0, 2009 (Matrix Science Ltd., London, UK) allowing the label-free analysis. They were searched against NCBInr_012014 (19922528 sequences; 6828478126 residues) with Rattus taxonomy selection (61059 sequences) using Mascot engine v.2.3.01 (Matrix Science Ltd.), with carbamidomethylation as fixed modification (+57 Da), oxidation of methionine, tryptophan and histidine (+16 Da), acetylation of lysine (+42 Da), methylation of lysine and arginine (+14 Da) and phosphorylation of serine and threonine (+80 Da) as variable modifications, one trypsin missed cleavage and a tolerance of 0.1 Da for both precursor and fragment ions.

**Proteome data analysis**: The UniProtKB ID Mapping tool available on the UniProtKB web site (www.uniprot.org) was used to obtain the ID numbers of the proteins of interest. The ExPASy website (http://web.expasy.org/compute_pi/) was used to compute the theoretical isoelectric point (Ip) and molecular weight (MW). We used the GENEmania system (http://www.genemania.org) to identify biological functions in which the proteins of interest are involved. The QuickGo website (http://www.ebi.ac.uk/QuickGO) and Panther (http://pantherdb.org/) were used to represent schematically the biological functions identified and to localize the main biological functions. Subsequently, the Cytoscape software was used to integrate this large amount of information, facilitating the analysis work. 

**Western Blot**: Twenty-four animals were used in this experiment (6/group). Western blot was used to confirm the results obtained with 2-D electrophoresis proteomics. Hippocampal samples (*n* = 6/group) were homogenized in lysis buffer and quantified using the Bradford method [63]. SDS-PAGE electrophoresis gels were used for protein separation. After the electrophoretic run, protein bands were transferred by electro blotting to polyvinylidene fluoride (PVDF) membranes, and incubated with primary antibodies (anti-PKM2, Anti-Dpysl2, Anti-Pdhb and Anti-Pebp1). In the sequence, membranes were washed, incubated with the appropriated secondary antibody and subjected to immunodetection procedure by chemiluminescence. Then membranes were washed and incubated with anti-β-actin. Bands were quantified by optical densitometry and the analysis was performed using the ratio between the protein of interest and the corresponding loading control (antibody raised against β-actin) for standardization of data.

**Statistical analysis**: The PDQuest software generated statistical difference of optical density in the proteomic study. Western blotting data was analyzed by Analysis of Variance (ANOVA). The post-hoc Tukey test was used when appropriate to identify statistical significance. Both, ANOVA and post-hoc Tukey were performed with 10,000 bootstrap resampling. Statistical analysis was performed in R version 3.0.2 [64].

## 5. Conclusions

Looking for long-term alterations in the hippocampal proteome, we observed a global reduction of glycolysis- and respiration-related proteins, but also of proteins related to encapsulation and neurotransmitter release (Hspa8 and Snca), neuronal excitability (Adam17), and axonal vesicle transport (Tubb2c). We observed alterations in the TCA cycle, which may impact on the synthesis of glutamate, GABA and aspartate [65]. In addition, we found alterations in proteins related to neuronal development and plasticity (Dpysl2, Gap43 and Arhgdia). It is noteworthy that many proteins affected are linked to glucose metabolism. Several studies have highlighted the anticonvulsant effect related to alterations in glucose metabolism and the ketogenic diet has been the target of these studies. The ketogenic diet changes several mechanisms related to cellular metabolism, mainly by deviating glucose metabolism as the main energy source of the cell and activating anticonvulsive and neuroprotective mechanisms [60]. On the other hand, it is not surprising that a reduction of brain reorganization, as seen after CRS treatment in the LiPilo model [8]), will largely involve mechanisms linked to development and plasticity. Further studies will be necessary to determine whether these altered proteins are caused by the epileptic condition or whether they participate in the process of epileptogenesis.

## Figures and Tables

**Table 1:** Proteins differentially expressed in the hippocampus of the four groups of rats.

Protein	Gene	UniProt ID	Mean of Optical Density			
			CT	DZP	CRS-TLE	CRS-NCS
Aconitase	Aco2	Q99798	4936	7904	6626	453 * ^# §^
ADAM metallopeptidase domain 17	Adam17	Q9Z0F8	1378	1448	1380	911 * ^# §^
Aldolase A	Aldoa	P05065	7537	9228	9117	679 * ^#^
Aldolase C	Aldoc	P05063	2136	2659	1381 ^#^	1951 ^#^
Alpha internexin	Ina	P23565	8882	6997	5493 *	6656
Alpha synuclein	Snca	P37377	4141	1868	657 *	5590 ^# §^
ATP synthase alpha subunit	Atp5a1	P15999	9684	9106	5389	764 * ^#^
ATP synthase delta subunit	Atp5d	P35434	2707	2437	2597	4079 * ^# §^
Creatine kinase U-type	Ckmt1	P25809	5151	5242	4467	3405 * ^#^
Dihydropyrimidinase-like 2	Dpysl2	P47942	2079	6635 *	3166 ^#^	2962 ^#^
Enolase 4	Eno4	D3ZRT2	843	493	723	1168 ^# §^
Enolase 2	Eno2	P07323	12,747	13,264	20,937 * ^#^	12,009 ^§^
Glyceraldehyde 3-phosphate dehydrogenase (Similar)	RGD1565368	P04797	8984	9174	8824	3971 * ^# §^
Glyceraldehyde 3-phosphate dehydrogenase	Gapdh	P04797	21,062	26,009	23,227	6032 * ^# §^
Guanine nucleotide-binding protein G(i), alpha-1 subunit	Gnai1	P10824	8054	2625 *	3925 *	2459 *
Heat shock cognate 71 kDa protein	Hspa8	P63018	4763	13,475 *	7216 ^#^	3475 ^#^
lactate dehydrogenase chain-B	Ldhb	P42123	7183	7058	2502 * ^#^	10,166 * ^# §^
Myosin-6	Myh6	P02563	3063	3814	4148	1223 * ^# §^
Neuromodulin	Gap43	P07936	4202	10,646 *	20,957 * ^#^	7264 ^# §^
Phosphatidylethanolamine binding protein *1*	Pebp1	P31044	9292	4951 *	4854 *	9429 ^# §^
Pyruvate dehydrogenase E1 component	Pdhb	P49432	3930	2253 *	2647	7687 * ^# §^
Pyruvate kinase M1/M2	Pkm2	P11980	7605	8491	7945	3497 * ^# §^
Rho GDP dissociation inhibitor *1*	Arhgdia	Q5XI73	2987	2383	5142	767 * ^# §^
Tubulin beta-2C chain	Tubb2c	P69897	127,231	142,920 *	98,280 * ^#^	48,681 * ^#^

Statistics: * (*p* < 0.05) = difference from CT group; ^#^ (*p* < 0.05) = difference from DZP group; ^§^ (*p* < 0.05) = difference from CRS–TLE group.

**Table 2:** Percentage of proteins related to protein class, cell compartment, molecular function and biological function in the comparison between groups.

**Protein Class**	**CT vs. DZP**	**CT vs. CRS-NCS**	**CT vs. CRS-TLE**	**DZP vs. CRS-NCS**	**DZP vs. CRS-TLE**	**CRS-NCS vs. CRS-TLE**
transporter		6%		5%		
hydrolase		6%		11%	33%	
oxidoreductase	25%	18%	14%	16%	33%	21%
cell junction protein		6%		5%		7%
enzyme modulator	25%	18%	14%	11%		14%
lyase	25%	12%	14%	11%	33%	21%
transferase	25%	12%		11%		7%
nucleic acid binding		6%		5%		
cytoskeletal protein		12%	14%	11%		7%
signaling molecule		6%		5%		7%
chaperone			14%	5%		7%
structural protein			14%			
membrane traffic protein			14%	5%		7%
**Cell Compartment**	**CT vs. DZP**	**CT vs. CRS-NCS**	**CT vs. CRS-TLE**	**DZP vs. CRS-NCS**	**DZP vs. CRS-TLE**	**CRS-NCS vs. CRS-TLE**
synapse	17%		13%	8%	33%	14%
membrane	17%	17%	13%	8%		14%
macromolecular complex	17%	25%	13%	17%		
cell part	33%	33%	38%	33%	33%	29%
organelle	17%	17%	25%	25%	33%	29%
cell junction		8%		8%		14%
**Molecular Function**	**CT vs. DZP**	**CT vs. CRS-NCS**	**CT vs. CRS-TLE**	**DZP vs. CRS-NCS**	**DZP vs. CRS-TLE**	**CRS-NCS vs. CRS-TLE**
binding	25%	20%	17%	17%		17%
receptor activity		5%		6%		
structural molecule activity		10%	17%	11%		8%
signal transducer activity	25%	5%	17%			
catalytic activity	50%	50%	50%	56%	100%	67%
transporter activity		10%		11%		8%
**Biological Function**	**CT vs. DZP**	**CT vs. CRS-NCS**	**CT vs. CRS-TLE**	**DZP vs. CRS-NCS**	**DZP vs. CRS-TLE**	**CRS-NCS vs. CRS-TLE**
response to stimulus	14%	5%	10%			
immune system process	14%			5%	20%	
developmental process		5%	30%	5%		7%
cellular process	14%	25%		24%		27%
multicellular organismal process		5%		5%		7%
Metabolic process	29%	35%	30%	33%	60%	40%
biological regulation	14%	5%	10%		20%	7%
cellular component organization or biogenesis	14%	10%	10%	14%		13%
localization		10%	10%	14%		

**Table 3:** Percentage of proteins involved in each specific pathway for each relevant group comparison.

Pathway	CT vs. DZP	CT vs. CRS-NCS	CT vs. CRS-TLE	DZP vs. CRS-NCS	DZP vs. CRS-TLE	CRS-NCS vs. CRS-TLE
ATP synthesis (P02721)		3%		4%		
Metabotropic glutamate receptor group III pathway (P00039)	5%	3%	5%			
Apoptosis signaling pathway (P00006)	5%			4%	14%	
Opioid proopiomelanocortin pathway (P05917)	5%	3%	5%			
Alzheimer disease-amyloid secretase pathway (P00003)				4%		6%
Alzheimer disease-presenilin pathway (P00004)		3%		4%		6%
Opioid prodynorphin pathway (P05916)	5%	3%	5%			
Alzheimer disease-amyloid secretase pathway (P00003)		3%				
Opioid proenkephalin pathway (P05915)	5%	3%	5%			
Enkephalin release (P05913)	5%	3%	5%			
Inflammation mediated by chemokine and cytokine signaling pathway (P00031)	5%	6%	5%	4%		6%
Dopamine receptor mediated signaling pathway (P05912)	5%	3%	5%			
Parkinson disease (P00049)	5%		5%	8%	14%	6%
EGF receptor signaling pathway (P00018)	5%		5%	4%		6%
Gonadotropin-releasing hormone receptor pathway (P06664)	5%	3%	5%			
PI3 kinase pathway (P00048)	5%	3%	5%			
Cytoskeletal regulation by Rho GTPase (P00016)		6%		8%		6%
Nicotine pharmacodynamics pathway (P06587)	5%	3%	5%			
Notch signaling pathway (P00045)		3%		4%		6%
Nicotinic acetylcholine receptor signaling pathway (P00044)		3%		4%		6%
Muscarinic acetylcholine receptor 2 and 4 signaling pathway (P00043)	5%	3%	5%			
Metabotropic glutamate receptor group II pathway (P00040)	5%	3%	5%			
Fructose galactose metabolism (P02744)		3%		8%	14%	
Huntington disease (P00029)		6%		8%		6%
Pyruvate metabolism (P02772)		3%		4%		6%
Wnt signaling pathway (P00057)		3%		4%		
Heterotrimeric G-protein signaling pathway-Gi alpha and Gs alpha mediated pathway (P00026)	5%	3%	5%			
5HT1 type receptor mediated signaling pathway (P04373)	5%	3%	5%			
Glycolysis (P00024)		6%	5%	15%	29%	19%
GABA-B receptor II signaling (P05731)	5%	3%	5%			6%
Endogenous cannabinoid signaling (P05730)	5%	3%	5%			
TCA cycle (P00051)		3%		4%		6%
FGF signaling pathway (P00021)	5%		5%	4%		6%
Axon guidance mediated by semaphorins (P00007				4%	14%	
Pyrimidine Metabolism (P02771)				4%	14%	
